# The Clinical Status of Stem Cell Therapy for Ischemic Cardiomyopathy

**DOI:** 10.1155/2015/135023

**Published:** 2015-05-26

**Authors:** Xianyun Wang, Jun Zhang, Fan Zhang, Jing Li, Yaqi Li, Zirui Tan, Jie Hu, Yixin Qi, Quanhai Li, Baoyong Yan

**Affiliations:** ^1^Cell Therapy Laboratory, The First Hospital of Hebei Medical University, Shijiazhuang, Hebei 050000, China; ^2^Department of Immunology, Basic Medical College, Hebei Medical University, Shijiazhuang, Hebei 050017, China; ^3^Department of Breast Center, The Fourth Hospital of Hebei Medical University, Shijiazhuang, Hebei 050011, China; ^4^Department of Thoracic Surgery, The Fourth Hospital of Hebei Medical University, Shijiazhuang, Hebei 050011, China; ^5^School of Nursing, Hebei Medical University, Shijiazhuang, Hebei 050000, China; ^6^Department of General Surgery, The Fourth Hospital of Hebei Medical University, Shijiazhuang, Hebei 050011, China

## Abstract

Ischemic cardiomyopathy (ICM) is becoming a leading cause of morbidity and mortality in the whole world. Stem cell-based therapy is emerging as a promising option for treatment of ICM. Several stem cell types including cardiac-derived stem cells (CSCs), bone marrow-derived stem cells, mesenchymal stem cells (MSCs), skeletal myoblasts (SMs), and CD34^+^ and CD 133^+^ stem cells have been applied in clinical researches. The clinical effect produced by stem cell administration in ICM mainly depends on the transdifferentiation and paracrine effect. One important issue is that low survival and residential rate of transferred stem cells in the infracted myocardium blocks the effective advances in cardiac improvement. Many other factors associated with the efficacy of cell replacement therapy for ICM mainly including the route of delivery, the type and number of stem cell infusion, the timing of injection, patient's physical condition, the particular microenvironment onto which the cells are delivered, and clinical condition remain to be addressed. Here we provide an overview of the pros and cons of these transferred cells and discuss the current state of their therapeutic potential. We believe that stem cell translation will be an ideal option for patients following ischemic heart disease in the future.

## 1. Introduction

Reduced blood supply in infracted myocardium is the leading cause of morbidity and mortality in patients with ischemic cardiomyopathy (ICM) [1, 2]. While approximately 1% of adult cardiomyocytes possess ability of self-renewal, they cannot afford heart tissue impairment from serious or acute myocardial infarction [3–5]. Thus, ischemia-induced cardiomyocyte apoptosis and necrosis damage left ventricle geometry undergoing progressive ventricle remodeling, hypertrophy, and fibroblast proliferation resulting in scar information and poor contractility of left ventricle [6–8]. The common treatment strategies such as pharmacotherapy, coronary artery bypass grafting (CABG), and coronary artery stent enable the recovery of blood supply to the ischemic regions and relatively alleviate pain and suffering, but they fail to treat the pathophysiological changes following ischemic injury and regenerate novel muscle tissue. Therefore, the ideal treatment effect is to make myocardial cell regeneration resident cardiac progenitor cells or other exogenous multipotent stem cells [9]. Stem cell implantation treatment for ICM has brought a new dawn for patients while it faces a new challenge. Accumulating evidences have reported that stem cells repaired damaged heart by the means of differentiation to cardiac muscle cell, promoting angiogenesis, forcing proliferation of endogenous cardiac stem cells, and secreting cytokines, chemokines, and growth factors to activate endogenous reparative responses, inhibit cell apoptosis and fibrosis, and improve myocardial contraction [10]. In the last decade, many clinical trials have been implemented to assess the safety, feasibility, and efficacy of stem cell administration in patients with ischemic cardiomyopathy. Different cell types including bone marrow-derived stem cells, mesenchymal stem cells (MSCs), cardiac-derived stem cells (CSCs), skeletal myoblasts (SMs), and hematopoietic stem cells (HSCs) have been used to evaluate the cell-based therapeutic potential. However, promising results from most clinical studies to improve functional parameters have yielded to the few mixed ineffective treatments. Delivery modalities, cell types and dose, cell isolation procedures, and timing of cell transplantation may determine the curative effect on cardiac functional recovery [11–13]. Here, the current status of clinical research and future outlook of stem cell-based therapeutics for ischemic cardiomyopathy are elaborated.

## 2. Types of Stem Cell and Their Clinical Studies

In the last two decades, many different stem cell populations have been investigated and suggested to enhance cardiac function recovery in clinical trials. These stem cells can be categorized according to their cellular structure, function, origin, or cell surface marker, transcription factor, and specific protein. The simplest and most common way to group them depends on their site of origin. Stem cells isolated from heart are named cardiac-derived stem/progenitor cells and other types of stem cells are known as extracardiac-derived stem cells. Here, recent clinical trials of stem cell replacement therapy for ICM are described in Table 1.

### 2.1. Cardiac-Derived Progenitor/Stem Cells

In 2003, cardiac stem cells (CSCs) were first discovered by Nadal-Ginard and colleagues [14] which break the traditional idea that heart was terminal differentiated organ. The multipotent and self-renewing characteristics of these cells have been identified in animal models which showed their ability to give rise to cardiomyocytes, endothelial cells, and smooth muscle cells indicating a potential regenerative capacity of adult heart [15]. The experimental studies have reported that cardiomyocyte lineages can be derived from different types of cardiac stem cells including c-kit^+^ cell, isl-1^+^ cell and sca-1^+^ cell (restricted to murine hearts), and cardiosphere-derived cells (CDCs) [16]. CDCs are isolated from cardiac biopsies and grow as self-adherent clusters containing a heterogenous cell population of stem cells positive for c-kit (endogenous CSCs), CD105, and CD90 (cardiac MSCs) but negative for CD 45 (hematopoietic stem cell) indicating their capacities of clonogenic, self-renewal, and multilineage differentiation [17]. Accumulating evidences have described the capabilities of CSCs or CDCs in regenerative ability of cardiac muscles without teratoma formation [16, 18].

CSCs residing in the myocardial inches are mainly located in left ventricular apex and atrial tissue. Although limited number of CSCs, 1 CSC per 8,000 to 20,000 cardiomyocytes, can generate enough cardiomyocytes a day to maintain the balance of the renewal of myocardial cells in healthy heart, they cannot bear great or acute damage of cardiac tissue. The reasons for the limitations of CSCs include the following aspects: the number and viability of CSCs decrease with age and further decrease in coronary heart disease [19–22] and c-kit^+^ CSCs nearly lost their homing ability in postnatal heart [23]. Therefore, exploiting practical and effective methods for the isolation and expansion of CSCs will be great impetus for CSC therapy for ischemic heart disease. It is exciting that Smith and colleagues have presented a feasible and safe method for the isolation and expansion of adult CSCs from endomyocardial biopsy specimens [24] which provided possibility to treat ICM.

To date, several clinical trials have been allowed and completed to display the feasibility, safety, and efficacy of CSCs translation therapy in patients with ICM. The first clinical phase I trial recruiting 16 patients with CABG and postinfarction left ventricular (LV) dysfunction (EF ≤ 40%) suggested that intracoronary infusion of 1 million autologous CSCs (c-kit^+^) increased LVEF from 30.3% to 38.5% at 4 months and 42.3% at 1 year and decreased infract size in seven patients from 32.6 g to 24.8 g at 4 months (*P* = 0.004) and 22.8 g at 1 year (*P* = 0.04) which revealed a striking treatment potential of CSCs [25]. Thereafter, a randomized phase I trial conducted by Makkar assessed the safety of intracoronary infusion of CDCs in patients with LV dysfunction after myocardial infarction (MI) (LVEF = 25%~45%) [26]. The corresponding results showed reduced scar mass (*P* = 0.001), increased viable heart mass (*P* = 0.01), and regional contractility (*P* = 0.02) at 6 months in CDC treatment group, but there are no different changes in end-diastolic volume, end-systolic volume, and LVEF comparing with standard care patients. Fortunately, no serious adverse effect occurred by 6 months indicating the safety of CDC implantation treatment of MI. Recently, a randomized and controlled CADUCEUS trial (cardiosphere-derived autologous stem cells to reverse ventricular dysfunction) was carried out in patients with LV dysfunction to examine the efficacy of this therapeutic method [27]. The major results suggested that CDC treatment had a significant relationship with the improvement of LVEF and reduction of scar size at 1 year after treatment comparing with routine-care control patients [27]. By now, CSC therapy mostly focus on the patients with MI and LV dysfunction, mainly due to the cardiac fibrosis with no blood supply in the infraction location and this therapy showed more efficacy in MI than chronic ICM. However, these limited clinical results cannot fully prove the practical competence of CSC therapy for ICM. Although these clinical data may not satisfy every patient and clinical investigator, they provide important basis for exploiting more effective cell processing methods and have initiated larger-scale phase I or II clinical studies (NCT01458405 and NCT01758406) to evaluate the curative effect of CDC/CSC transplantation into patients with ICM.

### 2.2. Extracardiac-Derived Stem Cells

#### 2.2.1. Bone Marrow Mononuclear Cells

Bone marrow mononuclear cells (BMMNCs) are a mixed population of various types of undifferentiated cells containing primary early committed cells, 2%~4% hematopoietic stem cells (HSCs)/endothelial progenitor cells (EPCs), and a fraction part of approximately 0.01% MSCs [55]. Although most of BMMNCs are not stem cells, the cardiac repair effect is considered to mainly depend on the significant source of HSCs which contribute to the development of new cardiac tissue and blood vessels. It is commonly accepted that the cardiac repair by HSCs therapy may be largely dependent on secretion of growth factors and other proteins to promote angiogenesis [56, 57] and stimulate cell proliferation and migration of endogenous cardiac stem cells or cardiomyocytes [11, 58].

A case study that referred to one patient with MI first demonstrated the therapeutic possibility under clinical conditions using intracoronary transplantation of autologous BMMNCs [59]. Afterwards, several clinical trials proved that intracoronary administration of adult bone marrow stem/progenitor cells (BMCs) in patients with acute myocardial infarction (AMI) improved LV contractile function and depressed LV remodeling which have been reported by REPAIR-AMI trials [41, 60, 61] and TOPCARE-AMI trial [42]. In addition, treatment with BMCs significantly reduced adverse clinical events [41, 62]. Therefore, the clinical experimental responses confirm the feasibility and possibility for the BMMNC infusion therapy for ICM though the detailed and solid action mechanisms of BMMNCs have not been concluded. Subsequently, more convincing evidences about the improvement of LVEF, cardiac contractility, and exercise capacity were supported by numerous larger-scale clinical trials.

To date, the largest meta-analysis reviewed by Jeevanantham compiled and systematically evaluated the clinical data from 50 studies enrolling 2625 patients with acute or chronic ICM [63]. Regardless of the detailed design of this study (e.g., path and timing of delivery, number of administrated cells, follow-up time after cell fusion, various populations of BMMNC, evaluation parameter, and imaging modalities) and the specific type of ICM (AMI or chronic ICM), BMMNC-treated patients showed a modest increase of LVEF (~3.96%) and smaller reduction of infarct size (~4.03%), LV end-systolic volume (~8.91 mL), and LV end-diastolic volume (~5.23 mL) compared to control subjects. This analysis also exhibited that BMMNC administration significantly reduced occurrence of some events including mortality, and the recurrent rate of MI and stent thrombosis. This modest improvement was consistent with another recent meta-analysis published in 2014 [64]. These pooled results from 32 trials comprising 1300 BMMNC-treated patients and 1006 control-treated patients both with acute coronary syndrome or stable coronary disease demonstrated statistically significant increase in LVEF (~4.6%) and decrease in perfusion defect size (~9.5%) without influence of baseline ejection fraction or perfusion defect size. Therefore, these outcomes of statistical analysis support the BMMNC therapy for MI to improve cardiac infarction, cardiac remodeling, and patient-important clinical outcomes. However, another recent meta-analysis drew a discrepancy conclusion from 22 randomized controlled trials [65]. In this study, compared with control arm, BMMNC therapy was safe on the whole in patients with ICM but neither promoted the recovery of heart function on MRI-derived parameters nor improved clinical outcome despite slight increase in LVEF (~2.1%). The lack of ventricular improvement by BMMNC therapy was paralleled in a more recent clinical trial recruiting 28 patients with advanced ICM [40]. After 6 months, BMMNC-treated patients showed no significant improvement in LVEF, LV end-systolic volume (LVESV), and LV infarct volume indicating ineffective outcome of this cell therapy project. Another new placebo-controlled study including 65 patients with ICM and LVEF < 50% displayed no statistically significant increase in LVEF and reduction in infarct size as a percentage of LV mass in BMMNC-treated group [30]. By now, it is apparent that BMMNC therapy provided highly variable and inconsistent results which showed mild to modest or no benefit effects in LV function and geometry [43]. Fortunately, no major adverse events were detected in all of the trial participants. On the whole, BMMNC therapy has been widely used in patients with the acute or chronic ICM and showed its importance in the therapy for ICM. However, the discrepant results have come out which may be due to numerous factors mainly including the method of BMMNC isolation and the type and total number of injected cells. The cellular mechanisms affecting the therapy efficacy mainly include efficient homing, proliferation, engraftment, and differentiation of infused BMMNCs which can be influenced by various culture conditions. A large-scale randomized multicenter European clinical trial is currently recruiting AMI patients to investigate intracoronary infusion of BMMNCs for assessing all-cause mortality (NCT01569178 and NCT01693042). It is expected that these data will be launched for larger randomized controlled clinical studies which involve the improvements in cell preconditioning, cell isolation procedure, and selection plan of cell infusion.

#### 2.2.2. Mesenchymal Stem Cells

MSCs mainly express cell surface antigens of CD73, CD90, CD105, CD44, CD106, and CD166 and negatively express CD34, CD31, CD14, CD45, CD133, and CD105 surface molecules [66, 67]. They have advantages of self-renewal ability, multilineage differentiation potential, low immunogenicity, immunosuppressive properties, and low tumorigenicity [68] and face no risk of immune rejection by the host for lacking expression of major histocompatibility complex class II [69]. Under proper stimulation MSCs can differentiate into cardiomyocytes or secrete some growth factors for cardiac repair. However, the survival rate and migration ability in target tissue are two key factors to determine the therapeutic effect of MSCs. Most clinical studies showed improvement in cardiac function after MSC treatment of ICM despite of few ineffective results of MSC replacement therapy [70].

Earlier clinical studies showed positive effect of MSC treatment in ICM. A placebo-controlled trial recruited 69 patients with AMI after percutaneous coronary intervention (PCI) who were treated by intracoronary delivery of autologous MSCs [71]. Three months after transplantation comparing with control group, significant improvement of LVEF and ratio of end-systolic pressure to end-systolic volume indicated effective abilities of cardiac repair and reverse remodeling after autologous MSC administration [71]. Intravenous injection of allogeneic MSCs which reduced ventricular tachycardia episodes and increased LVEF within six months produced a similar effect to the above conclusion [72]. Later, a clinical trial of intramyocardial injection of autologous MSCs in patients with remote myocardial infarction for 1 year showed a decreased infarct size and improved regional LV function by test of peak Eulerian circumferential strain [43]. These conclusions were further confirmed by the latest clinical trials which suggested that patients following AMI showed improvement in LVEF in bone MSC-treated group versus control group [32, 73]. But paper published in JAMA in 2014 revealed that although transendocardial stem cell injection with MSCs improved 6-minute walk distance, regional myocardial function, and peak Eulerian circumferential strain at the site of injection and reduced infarct size as a percentage of LV mass, no changes were observed in left ventricular chamber volume and ejection fraction [30]. So it was suggested that either delivery methods or allogeneic/autologous stem cells are associated with the therapeutic effect. There were no serious adverse events emerging across these studies. Two meta-analyses have evaluated bone MSCs therapy for ischemic heart disease and concluded that bone MSC treatment significantly reduced mortality risk, decreased angina episodes per week, and leaded to an even better life quality [74, 75]. A recent power meta-analysis involving 1255 patients found moderate quality evidence that bone MSC treatment improved LVEF [76]. Therefore, most of these studies showed to some extent improvements in cardiac function and attenuation in left ventricular dilation and remodeling either in MI patients or ICM patients. Although the curative effect of MSC grafting did not reach the ideal target, considerable prospect has been demonstrated in the treatment of AMI or ICM. To be sure, all of these studies showed safety and no severe adverse events of MSC grafting in patients with MI or acute/chronic ICM diseases. Numerous ongoing clinical trials have been encouraged for further investigation of the safety and efficacy after MSC treatment in patients with ICM (NCT01556022, NCT01216995, and NCT00644410). What influences the efficacy of MSC therapy also needs to be explored and investigated.

#### 2.2.3. Skeletal Myoblasts

SMs are a small population of undifferentiated and inactive cells residing within mature skeletal muscle fiber. Once injury occurs, SMs are activated from static state and rapidly proliferate and differentiate into muscle fibers to replace injured or dying muscle cells. So a question is derived; whether SMs can fuse with cardiac muscle cells to promote regeneration of cardiac muscle needs to be further confirmed. Besides, SMs feature several unique advantages such as autologous origin, being readily available, easy harvest, a high scalability potential, a high resistance to ischemia, and lack of tumorigenicity [77]. Experiment data has proved their ability to migrate into infarct region, differentiate into myotubes [78], and promote angiogenesis [79], thereby improving cardiac function and attenuating cardiac remodeling. These results pave the way for clinical research and application of SM replacement therapy for ICM. SMs were the first cell type to be employed in the clinical trials. Most of the studies mainly pointed out the safety and feasibility of SM infusion therapy [80, 81], but the efficacy is quite controversial. Several pilot studies showed to some degree improvement in LVEF and ventricular remodeling by SM fusion treatment. Patients with severe ICM injected with autologous SMs showed improvement of LVEF and New York Heart Association functional class after follow-up of 6 months or 11 months [82, 83]. Although these studies showed the feasibility, safety, and modest effect of SMs grafting in ICM, a high incidence of ventricular arrhythmias event after myoblast transplantation requires immediate attention. Besides, a recent study with about 6-year long-term follow-up after autologous SM transplantation in seven patients with ischaemic heart disease implied that SM engraftment did not improve LV function [54]. Moreover, SM-treated group had higher incidence of interventions in patients fitted with an internal cardioverter defibrillator compared with control group (87% and 13%, resp.) [84]. Thus, SM therapy has shown a more certain therapeutic effect in the patients with chronic ICM than that of acute ICM which will appeal for many scientists to explore the therapeutic effect of SMs in AMI patients. Yet, these conflicting and vague results warrant initiation of larger randomized double-blind trials (NCT00526253) and engineered SMs should be exploited for further efficacy assessment in acute/chronic ICM. However, it is noteworthy that ventricular arrhythmias may be a potential risk during SM grafting treatment. At this time, SMs may not be a primary option for ICM/MI treatment.

#### 2.2.4. Other Types of Stem Cell

CD34^+^ cells and CD133^+^ cells are two distinct cell populations isolated from bone marrow or peripheral blood. Several clinical researches have been completed focusing on the cardiac repair by promoting angiogenesis and neovascularization in ischemic tissues [85]. Transendocardial injection of peripheral blood CD34^+^ cells into patients with ICM (LVEF < 40%) increased LVEF and 6-minute walk distance which demonstrated improved LV function and better exercise capacity [46]. Besides, CD34^+^ cell administration in patients with varying degrees of angina and myocardial ischemia showed significant improvements in angina frequency and exercise tolerance [44, 45, 86, 87]. Major adverse effects were not observed in these CD34^+^ cell therapy researches. These results emphasize the feasibility and safety of CD34^+^ cell therapy by delivery of either intramyocardial injection or intracoronary infusion and the efficacy has close contact with the number of transplanted cells. CD133^+^ cell engraftment has also been approved to have positive effect for modest improvement in cardiac function without severe adverse effect in patients with chronic ICM through intramyocardial injections [50, 51, 88, 89]. But, a clinical trial named Cardio133 proved no effect on LV function and clinical symptoms in patients with chronic ischemic heart disease and impaired LV function treated by intramyocardial injection of CD133^+^ BMCs [52]. Therefore, both of these two types of stem cells have shown more promising potential in chronic ICM treatment than AMI which deserve further investigations in the treatment of acute ICM. Overall, these results preliminarily show the feasibility and effectiveness of CD34^+^ or/and CD133^+^ cells engrafting therapy for ICM and present a promising alternative for cell grafting management in ICM patients. Several ongoing researches are expected to get more effective results (NCT00950274 and NCT01337011). Further studies are required to develop new technologies to treat ICM. Thus more preclinical and clinical trials are needed to elucidate the effects of stem cell infusion on the progression of heart repair. Numerous other types of stem cells will be exploited to evaluate their practical abilities to treat ICM in the future.

## 3. Influence Factors of Stem Cell Therapy for ICM

By now, it is apparent that the current state of stem cell therapy is quite promising but faces severe situations. Firstly, most clinical results show us the benefit effect of stem cell grafting for cardiac repair regardless of the cell types and delivery ways. Secondly, the current results of stem cell therapy for ICM with high variability and diversity display mild to modest or even conflicting results in LV function and geometry and short-lived duration of cardiac improvement. Lastly, some stem cell types and delivery pathways displayed severe adverse effects such as cardiac arrhythmia and vascular restenosis which draw immediate attention of scientist and clinician. Many important factors affecting the efficacy of stem cell infusion in patients with ICM and MI mainly involve the following aspects: the delivery path of injection, the type and number of donor cells, the viability of fusion cells, and also the medical condition of patient. The delivery of stem cells is one of the most important factors to influence the curative effect which has been reviewed by some academics in detail [90–92]. Therefore, our discussion focuses on the other influence factors except delivery.

### 3.1. The Number and Phenotype of Stem Cells

The phenotype of injected cells has much correlation with the safety and efficacy. At present, MSC transplantation in ICM patients is yielding more promising results and fewer adverse effects versus SM and is easier to be acquired versus CSC. Random-effects meta-analysis performed on 888 animals through 52 studies suggested that BMMNC therapy showed less effect than MSC treatment. Sensitivity analysis described that efficacy was more relevant to higher cell number (≥10^7^) and late injections (>1 week) [93]. A recent comparative clinical trial supported more efficacy in MSC-treated group rather than BMMNC-treated group [30]. Besides, the number of cells injected in patients with varying degrees of ICM varies from study to study. One clinical trial has shown that low-dose concentration MSCs (20 millions) resulted in greater reduction in LV volumes and increase in LVEF compared to high-dose groups (100 million and 200 million) [29], which was consistent with another comparison results that low-dose intramyocardial injection of CD34^+^ cells (1*∗*10^5^ cells/kg) had greater improvement in exercise tolerance and lower weekly angina frequency at both 6 months and 12 months than that of high-dose group (5*∗*10^5^ cells/kg) [45]. These instances show the importance of the nature and dosage of injected cells which need further grope and exploration in more detailed clinical studies in the future.

### 3.2. Isolation Procedure

Cell preparation technologies are basically mature but to some extent variable because of the different incubation periods for screening and expansion. BMMNCs are readily available and injected into patients on the same day of harvest but other types of stem cells experience a process of selection, culture, and expansion which would take several days or weeks. Common methods of MSCs and CSCs in flasks involve a large number of open procedures and require prolonged culture times. Different centrifugation speed and washing buffer composition during the cell processing correlate much with therapeutic effect of ischemic heart disease [94]. Different protocols and regent components for cell isolation and storage held great distinctions in response to the functional capacity of blood-flow-recovery after BMMNC transplantation [95]. Red blood cell contamination during BMMNC isolation process also reduced cell viability, migratory function, colony-forming unit capacity, and neovascularization capacity [96]. A novel method, named Quantum Cell Expansion System, has been developed to generate higher numbers of MSCs in less time and at lower passages leading to a substantial reduction in pollution risk comparing with expansion in the flasks [97]. Thus, studies on establishing new technologies are underway to improve cell quality and quantity. Combination of preclinical and clinical studies will produce great amazing results in developing the best and simplest way to generate ideal stem cells.

### 3.3. Other Influence Factors

In addition to the aspects above, the cell activity, the injection time, the period of follow-up, and patient's physical differences are also relevant to the therapeutic effectiveness of cell-based treatment. Infusion time varies from several days to several weeks after AMI. A study of infusion timing showed no differences in LV function improvement at 4 months after intracoronary infusion of BMMNCs at either 5 to 7 days or 3 to 4 weeks among 200 patients with ST-segment elevation myocardial infarction comparing with control group [39]. The TIME randomized trial showed no significant improvement in global or regional EF in patients with left ventricular dysfunction after intracoronary infusion of 150 × 10^6^ BMMNCs at either 3 days or 7 days [98]. However, a pooled analysis of 7 randomized controlled trials enrolling 660 patients with AMI stated that bone MSC infusion at 4 to 7 days after AMI held greater improvement in LVEF and reduction of LV end-systolic dimensions compared to cell infusion within 24 hours which might link to the occurrence of acute inflammatory reactions immediately after AMI [99]. Besides, five-year follow-up study of intracoronary BMMNC infusion in 8 patients with stable severe ICM indicated no significant change in systolic and diastolic function [100], but 7-year follow-up of the DanCell study proved a beneficial effect of bone marrow-derived stem cells engrafting in patients suffering from chronic ischemic heart failure [31]. The cardiac improvement was also associated with the seriousness of patient's condition. A better improvement occurred in the patients with greater degree of segmental left ventricular dysfunction after transendocardial injection of MSCs [33]. Besides, it has also been proved that CDCs derived from heart failure patients displayed a greater therapeutic benefit than those from MI hearts and healthy hearts [101]. Therefore, cell replacement therapeutics is a complex process with many comprehensive elements. The way to achieve the best outcome requires optimum balance conditions of these influence factors.

## 4. Low Survival and Residential Rate of Transferred Stem Cells in the Infarcted Myocardium and Strategies for Increasing Efficacy

Although stem cell therapy is emerging as a promising approach to benefit cardiac healing and prevent cardiac heart failure after MI, the low efficacy issue still deserves further concern. That is because of not only significant loss of transplanted cells due to blood circulation, myocardial contraction, and leakage from the injection site but also low survival rate of resident cells due to harsh environment resulting from ischemic, hypoxia, oxidative stress, or inflammatory response [90]. A variety of methods have been emerging to improve cell survival rate and enhance functional capacity of these stem cells for promising application. The improvement projects include cell pretreatment by environment conditions and pharmacological, genetic manipulation prior to stem cell delivery, cotransplantation of stem cells with extracellular matrix molecules, nanofibers, hydrogels, or fibrin glues, and combination therapy using two types of stem cells.

Firstly, preconditioned cells can maintain a standby state by activating the cell survival signaling pathways to make them resistant to hostile environment. Hypoxic or shock wave preconditioning have been demonstrated effectively to prevent extensive apoptosis of transplanted cells through several processes including modification of cell phenotype, secretion of various cytokines, and increasing the transcription and translation of antiapoptotic genes [102–105]. Preclinical studies have shown that pretreating MSCs with transforming growth factor-alpha, basic fibroblast growth factor, interleukin-1*β*, or transforming growth factor-*β* improved MSC-mediated myocardial protection and stimulated angiogenesis [106–108]. It has also been proved that lysophosphatidic acid and atorvastatin can rescue rat bone MSCs from hypoxic or hydrogen peroxide induced apoptosis [109–112]. Secondly, genetic engineering of MSCs has become a promising approach to improve the MSC performance to protect them from apoptosis, increase their retention rate, and enhance their ability of migration and differentiation and paracrine effect. Overexpression of prosurvival, antiapoptotic, proangiogenic genes or homing receptors has generated significant increase in the survival rate of MSCs and improved their migratory behavior which has been reviewed in detail by Park et al. [113] and Yin et al. [114]. Thirdly, codelivery of stem cells with extracellular matrix molecules, nanofibers, hydrogels, or fibrin glues could directly increase the retention rate of grafted cells and also promote cell survival and differentiation. Encapsulation of CSCs within matrix-enriched hydrogel capsules prevented cell death and boosted the retention of CSCs [115]. Two latest studies have reported that the transplantation of MSCs cultured on nanomatrix or cardiac fibroblast-derived 3D extracellular matrix can successfully increase the retention rate of MSCs and promote cell proliferation, adhesion, and migration which demonstrated the potential application in regenerative therapy for ICM [116, 117]. Another experimental study demonstrated the formation of thick stratum and angiogenesis of engrafted monolayered MSCs through paracrine pathways [118]. However, grafting of engineered sheet into patients has not been implemented in clinical study due to the unpredictable consequences of transplant rejection and arrhythmias, propelling further investigation for the future. Lastly, combined cell transplantation of autologous SMs and BMMNCs has been proved feasible and effective in patients with severe ICM without generating fatal arrhythmias and complications [53]. After combined transplantation of SMs and bone marrow cells, the patients showed significant improvement in cardiac function and angiogenesis and decreased fibrosis size [119]. Moreover, codelivery of human CSCs and human MSCs through intramyocardial infusion created a greater reduction of infarcted size, a stronger improvement in left ventricular contractility, and 7-fold enhanced engraftment of stem cells compared to each cell therapy group and placebo group which illustrated an important biological interaction between c-kit^+^ CSCs and MSCs [120].

Thus, these preclinical and clinical data provide an important basis for the deeper research direction in the improvement of life quality and cardiac function. Therefore, so many fuzzy and unknown fields deserve further exploration for developing promising treatment methods of ICM.

## 5. Conclusion and Perspective

To summarize, the safety and feasibility of stem cell replacement therapy in ICM patients have been widely investigated in numerous clinical studies. MSC or BMMNC replacement therapy has been widely studied in the patients with acute or chronic ICM and shown a certain therapeutic effect. At present, MSC therapy shows more promising results in MI patients and makes a good foundation for the clinical application in the near future. CSCs is an emerging stem type which have been mainly investigated in AMI patients, so it owns a more developing space in ICM patients. Although SMs, CD34^+^, and CD133^+^ have been grafted in patients with ICM, they show less application potential than MSC or CSC treatment. Similar results to the transplantation of these types of stem cells are modest or discrepant improvement. However, the inconsistent results of efficacy evaluation are not a surprise to us due to different techniques and doses of cells delivered via multiple routes and patients with different conditions. On the whole, MSCs and CSCs appear to have greater application potential from the results of comprehensive analysis. As to cell delivery routes, intramyocardial and intracoronary show more promising future than intravenous injection. The action mechanisms of administrated cells are mostly inclined to paracrine effect by releasing of cytokines, chemokines, and growth factors to inhibit cell apoptosis and fibrosis and activating endogenous regenerative system. Other elements need to be further investigated to achieve the maximum effect regarding the optimal separation process, ideal cell type, cell dose, timing and route of delivery, and clinical indications. While a large number of comparative analysis studies provide very important outcomes, they are still not enough to offer the best choice of therapeutic program. Larger-scale randomized clinical studies are underway to optimize the therapeutic effect. Moreover, other cell types like human umbilical blood MSCs, umbilical cord MSCs, and adipose tissue-derived MSCs show a good prospect in the application of ICM treatment according to large amounts of preclinical data. This detailed summary will stimulate profound interest in pursuing additional and larger-scale clinical trials. We believe that through continuing and collaborative efforts multipotent stem cells transplantation will produce a satisfactory reply to ICM patients.

## Figures and Tables

**Table 1:** Stem cell-based clinical studies in patients with ICM since 2010.

Study	Comparators	NO.	Condition	Delivery	Main results	Overall evaluation
CSCs						
SCIPIO trial (2011) [25]	Auto CSCs versus control	16	EF ≤ 40%, phase I	IC	LVEF ↑, infarct size ↓	Improvement in LV systolic function, efficacy
CADUCEUS trial (2012) [26]	Auto CDCs versus standard care	31	MI, LVEF = 25–45%, phase I	IC	Scar mass ↓, viable mass ↑, regional contractility ↑, EDV-, ESV-, LVEF-, SAE-	Increase of viable myocardium, safe
SCIPIO trial (2012) [28]	Auto CSCs versus control	33	LVEF < 40%, phase I	IC	LVEF ↑, infarct size ↓, LV nonviable mass ↓, LV viable mass ↑, SAE-	Feasible, improvements in global and regional LV function
CADUCEUS trial (2014) [27]	Auto CDCs versus routine care	17	MI, phase I	IC	Scar size ↓, scar mass ↓, Viable mass ↑, SAE-	Safe and effective

MSCs						
Hare et al. (2012) [29]	Allo MSCs versus auto MSCs versus placebo	30	ICM, phase I/II	TED	Allo group: LVEDV ↓; Auto group: 6-minute walk distance ↑; both: EF-, MVO2-, SAE-	Improvements in LV function, life quality, and LV reverse remodeling
TAC-HFT (2014) [30]	Auto MSCs versus placebo	65	ICM, LVEF < 50%	TED	LV chamber volume-, LVEF-, 6-minute walking distance ↑, infarct size ↓, SAE-	Safety and modest efficacy
DanCell study (2014) [31]	Auto BMSCs versus baseline	32	Systolic dysfunction, LVEF 33 ± 9%	IC	Multivariate regression analysis, CD34(+) cell survival rate ↑, SAE-	Beneficial on chronic ischemic HF for long-term survival
SEED-MSC (2014) [32]	Auto BMSCs versus control	80	AMI	IC	LVEF ↑, SAE-	Safety
POSEIDON (2014) [33]	MSCs versus baseline	30	Chronic ICM	TED	EF ↑, SAE-	Scar size reduction and better ventricular function

BMMNCs						
STAR-heart study (2010) [34]	BMMNCs versus control	391	Chronic HF due to ICM, LVEF ≤ 35%	IC	LVEF ↑, exercise capacity ↑, oxygen uptake ↑, LV contractility ↑, long-term mortality ↓, SAE-	Improvements in LV performance, life quality, and survival
Hu et al. (2011) [35]	Auto BMMNCs versus placebo	60	Congestive HF due to severe ICM	Via CABG	LVEF ↑, LVESV ↓, wall motion index score ↑, 6-minutes walking distance ↑, SAE-	Efficacy, feasibility, and safety
Intrapatient comparison (2012) [36]	BMMNCs versus placebo	16	Chronic ICM	IM	Life quality ↑, LVESV ↓, LVEDV-, LVEF-, SAE-	Significant improvements in angina symptoms and MP
Antonitsis et al. (2012) [37]	Auto BMMNCs versus baseline	9	Severe ICM	CABG plus IM	LVEF ↑ at 3, 6, and 12 M, MP ↑ and infarct size ↓ at 6 and 12 M, SAE-	Feasibility and safety
FOCUS-CCTRN trial (2012) [38]	Auto BMMNCs versus placebo	153	Chronic ischemic HF, NYHA II–IV	TED	LVESV-, MVO2-, regional wall motion-, clinical improvement-, SAE-	No efficacy
Sürder et al. (2013) [39]	Auto BMMNCs versus placebo	200	STEMI	IC	LVEF-, SAE-	No efficacy
Heldman et al. (2014) [30]	Auto BMMNCs versus MSCs	65	ICM, LVEF < 50%, phase I/II	TED	6-minute walking distance ↑, infarct size ↓, LV chamber volume-, EF-, SAE-	Safety
END-HF (2014) [40]	Auto BMMNCs versus placebo	28	ICM, NYHA III-IV, LVEF < 40%	TED	LVEF-, LVESV-, LV infarct volume-, LV peri-infarct ischemic volume, SAE-	No improvements in LV function and remodeling, no efficacy

BMCs						
Assmus et al. (2010) [41]	BMCs versus placebo	204	Reperfused AMI	IC	Regional LV contractility ↑, SAE-	Reduction of major adverse events, LV function improvement
TOPCARE-AMI trial (2011) [42]	CPCs versus BMCs versus baseline	59	Reperfused AMI	IC	LVEF ↑, infarct size ↓, LVESV-, LVEDV ↑, SAE-	Long-term safety and efficacy
Williams et al. (2011) [43]	Auto BMCs versus baseline	8	MI	IM	EDV ↓, ESV ↓, infarct size ↓, SAE-	Improvements in LV regional contractility and reverse remodeling

CD34^+^ SCs						
Wang et al. (2010) [44]	Auto CD34^+^ SCs versus placebo	112	Intractable angina, CCSC III-IV	IC	Weekly angina episodes frequency ↓, exercise time ↑, MP ↑, SAE-	Safety and feasibility
Losordo et al. (2011) [45]	Auto CD34^+^ SCs versus placebo	167	Refractory angina	IM	Weekly angina frequency ↓ and exercise tolerance ↑ in the low-dose group, SAE-	Safety and efficacy
Poglajen et al. (2014) [46]	Peripheral blood CD34^+^ SCs versus medical therapy	31	ICM, phase I/II (LVEF < 40%)	TED	LVEF ↑, 6-minute walking distance ↑, N-terminal pro-B-type natriuretic peptide ↓, SAE-	Improvements in LV function and exercise capacity, efficacy

CD133^+^ SCs						
COMPARE-AMI trial (2010) [47]	Auto CD133^+^ SCs versus placebo	14	PCI and LVEF < 50%, phase II	IC	LVEF ↑, LV function ↑, MP ↑, SAE-	Safety and efficacy
COMPARE-AMI trial (2010) [48]	Auto CD133^+^ SCs versus placebo	20	AMI	IC	LVEF ↑, SAE-	Safety, feasibility, and efficacy
Kurbonov et al. (2013) [49]	Auto CD133^+^ SCs versus control	15	ICM and MI	IC	Scar size ↓, effort tolerance ↑, physical endurance ↑, overall autonomy ↑, SAE-	Safety, feasibility, and efficacy
IMPACT-CABG pilot trial (2013) [50]	Auto CD133^+^ SCs versus baseline	5	ICM, NYHA III	CABG plus IM	Systolic wall thickness ↑, the mean segmental wall thickness ↑, LVEF-, SAE-	Safety and feasibility
Assmann et al. (2014) [51]	Auto CD133^+^ SCs + CABG versus CABG	42	Severe ICM, LVEF = 15%–35%	CABG plus TEP	LVEF ↑, angina frequncy ↓, life quality ↑, SAE-	Improvement in myocardial function, safe and feasible
Cardio133 trial (2014) [52]	Auto CD133^+^ SCs versus placebo	60	Chronic ICM, LVEF < 35%	IM	6-min walking distance-, MLHFQ-, CCSC-, NYHA-, LVEF-, MP ↑, scar mass ↓, regional wall motion ↑, SAE-	No effects on global LV function and clinical symptoms

SMs						
Fujita et al. (2011) [53]	Auto SMs versus baseline	4	Severe ICM	IM	Two patients with brain natriuretic peptide levels ↓ and MP ↑, SAE-	Feasibility, only marginal improvements
Brickwedel et al. (2014) [54]	Auto SMs versus placebo	7	ICM, phase II	Via CABG	High-dosage group: LVEF-, LV volumes ↓; low-dosage group: LV volumes-; SAE-	No improvement in LV function, safety

SCs: stem cells; NO.: number of patients; BMSCs: bone mesenchymal stem cells; CPCs: circulating blood-derived progenitor cells; M: month; W: week; IC: intracoronary; IV: intravenous; IM: intramyocardial; TEN: transendocardial; TEP: transepicardial; LV: left ventricular; LVEF: left ventricular ejection fraction; PCI: percutaneous coronary intervention; HF: heart failure; CBFR: coronary blood flow reserve; MLHFQ: Minnesota Living With Heart Failure Questionnaire; STEMI: ST-elevation myocardial infarction; HRV: heart rate variability; LVAD: left ventricular assist device; CABG: coronary artery bypass graft; MV: myocardial viability; MP: myocardial perfusion; VCF: LV velocity of shortening; CFR: coronary flow reserve; Auto: autologous; Allo: allogeneic; NYHA: New York Heart Association class; CCS: Canadian Cardiovascular Society; CCSC: Canadian Cardiovascular Society class; WMSI: Wall Motion Score Index; 24-h Holter ECG: twenty-four-hour electrocardiographic monitoring; SAE: serious adverse events.
